# Functional Health Literacy: Psychometric Properties of the Newest Vital Sign for Portuguese Adolescents (NVS-PTeen)

**DOI:** 10.3390/nu13030790

**Published:** 2021-02-27

**Authors:** Osvaldo Santos, Miodraga Stefanovska-Petkovska, Ana Virgolino, Ana Cristina Miranda, Joana Costa, Elisabete Fernandes, Susana Cardoso, António Vaz Carneiro

**Affiliations:** 1 Instituto de Saúde Ambiental, Faculdade de Medicina, Universidade de Lisboa, 1649-028 Lisboa, Portugal; mpetkovska@medicina.ulisboa.pt (M.S.-P.); avirgolino@medicina.ulisboa.pt (A.V.); acmmiranda@gmail.com (A.C.M.); jfcosta@medicina.ulisboa.pt (J.C.); avc@medicina.ulisboa.pt (A.V.C.); 2 Unbreakable Idea Research, Lda., 2550-426 Painho, Portugal; 3 Faculdade de Medicina, Universidade de Lisboa, 1649-028 Lisboa, Portugal; elisabete.baratafernandes@gmail.com; 4 MARE, Escola Superior de Turismo e Tecnologia do Mar, Instituto Politécnico de Leiria, 2520-614 Peniche, Portugal; susana.cardoso@ipleiria.pt; 5 CiTechCare, Instituto Politécnico de Leiria, 2410-541 Leiria, Portugal; 6 Institute for Evidence Based Healthcare, 1649-028 Lisboa, Portugal; 7 Cochrane Portugal, 1649-028 Lisboa, Portugal

**Keywords:** functional health literacy, questionnaire, psychometrics, item response theory, adolescents

## Abstract

Self-management of health requires skills to obtain, process, understand, and use health-related information. Assessment of adolescents’ functional health literacy requires valid, reliable, and low-burden tools. The main objective of this study was to adapt and study the psychometric properties of the Newest Vital Sign for the Portuguese adolescents’ population (NVS-PTeen). Classic psychometric indicators of reliability and validity were combined with item response theory (IRT) analyses in a cross-sectional survey, complemented with a 3-month test-retest assessment. The NVS-PTeen was self-administered to students enrolled in grades 8 to 12 (12 to 17 years old) in a school setting. Overall, 386 students (191 girls) from 16 classes of the same school participated in the study (mean age = 14.5; SD = 1.5). Internal reliability of the NVS-PTeen was α = 0.60. The NVS-PTeen total score was positively and significantly correlated with Portuguese (*r* = 0.28) and mathematics scores (*r* = 0.31), school years (*r* = 0.31), and age (*r* = 0.19). Similar to the original scale (for the U.S.), the NVS-PTeen is composed of two dimensions, reading-related literacy and numeracy. Temporal reliability is adequate, though with a learning effect. IRT analyses revealed differences in difficulty and discriminative capacity among items, all with adequate outfit and infit values. Results showed that the NVS-PTeen is valid and reliable, sensible to inter-individual educational differences, and adequate for regular screening of functional health literacy in adolescents.

## 1. Introduction

Over the past decades, health literacy has become a flourishing field of research. This concept has evolved from a rather simple one, mainly focused on specific health- or disease-related contents, toward a complex construct [1]. Despite the variations concerning its definition, health literacy is generally understood as a set of individual capacities to obtain, process, and understand basic health information and services, which supports appropriate health- or disease-related decision making [2]. Health literacy relates to general literacy, as it involves the combination of verbal (reading and writing) and numerical skills with specific health literacy skills to access, understand, appraise, and apply the information gathered in order to make decisions and engage in health behaviors [1,3]. The extent to which an individual is autonomous and empowered to self-manage health, following the chain of access–understand–appraise–apply health-related information, is broadly indicated by his/her levels of functional, interactive, and critical literacy [4]. Individuals possessing basic reading, writing, and numeracy skills that are necessary for them to function effectively in everyday situations, including managing their health or disease more easily, show adequate levels of functional health literacy [1,4]. Increased autonomy and empowerment in self-management of health come from advanced cognitive, social, and literacy skills that allow individuals to gather, interpret, and apply health-related information to changing conditions (interactive literacy) [4] and, on top of this, critically appraise health-related information (critical literacy), promoting the individual’s control over his/her own health [4,5]. The ultimate consequences of adequate health literacy levels include better life course health outcomes and reduced burden of healthcare service utilization (e.g., costs, frequency, and length of hospitalizations) [1,6]. The potential positive effects of health literacy promotion have been increasingly debated and are increasingly influential as a policy priority by decision makers across countries (e.g., [7]). 

A critical step, prior to the design and implementation of sustainable and (cost- and time-) effective health literacy policies, is to measure citizens’ health literacy. The European Health Literacy Survey (HLS-EU), an eight-country effort to measure health literacy, reported inadequate health literacy for 12.4% of the adult population from the eight European countries that took part in the survey (Austria, Bulgaria, Germany, Greece, Ireland, the Netherlands, Poland, and Spain) [8]. Furthermore, 35% of the participants in the HLS-EU had limited health literacy. In the particular case of Portugal, two nationwide studies using the same HLS-EU instrument estimated that limited functional health literacy ranges between 49% [9] and 61% [10] for the Portuguese adult population.

Efforts to estimate health literacy have been mainly focused on clinical and community samples of adults (≥18 years old), whereas health literacy among children and adolescents has been sparsely measured [11,12,13,14,15]. Despite this, increased attention has been given to the association between parents’ health literacy levels and health outcomes of their dependents (e.g., [16,17]). At this level, the existing evidence suggests that parents’ limited health literacy is associated with a higher number of non-urgent visits to the pediatric emergency department [17] and hinders both their engagement in shared-decision making and their children’s therapeutic adherence [16]. As such, empowering children and adolescents to actively participate in health decision making in a meaningful manner is fundamental to achieving better health outcomes from a life course perspective. 

Health outcomes and behaviors in adolescence and adulthood are strongly associated. Thus, the lack of research in health literacy during adolescence is somehow striking. Adolescence is a life transition period characterized by numerous developmental changes (physical, cognitive, and emotional) (e.g., [18,19,20,21]), which are inherently linked to (health) behavioral change [20,22] and habit formation, as well as to an increasing level of individuals’ autonomy in many spheres of their lives, including health decision making. At this stage, several levels of influence—individual traits, peer and family influences, school and neighborhood environments—collectively determine individual development and the adoption of health behaviors [12], namely in relation to physical activity, healthy eating, substance abuse, and sexual risk-taking behaviors, these being major determinants of later life health outcomes and inequities [23,24,25]. Under this scenario, effective health literacy programs are expected to be implemented within the environments in which adolescents are embedded [12]. Schools are particularly relevant toward this end [26,27,28], as also emphasized by Manganello [12], because they provide the resources and supportive environment that facilitate the development of general literacy skills, i.e., reading, writing, and numeracy, which are also required for health literacy.

Given the undisputable role of health literacy during adolescence for health gains in a life span perspective, why are data scarce for this life stage? The lack of validated instruments for measuring health literacy, and, most specifically, functional health literacy, during adolescence is perhaps the main contributor to this knowledge gap (e.g., [14]). Much of the measurement instruments currently under use are of weak or moderate validity [29] and heterogeneous concerning their scope, which also shows the lack of consensus regarding the definition and conceptualization of health literacy, as previously mentioned [11,29,30]. Nevertheless, adaptations of health literacy measurement instruments developed for adults, such as the Test of Functional Health Literacy in Adults (TOFHLA) [31], the Rapid Estimate of Adult Literacy in Medicine (REALM) [32], and the Newest Vital Sign (NVS) [33] (just to name some of the most widely used tools in this area), have been used to assess functional health literacy in children and adolescents (e.g., [13,15,29]), although their psychometric properties have not always been studied. These three instruments measure different domains of functional health literacy: TOFHLA is a 50-item reading comprehension and numerical ability test that takes approximately 22 min to complete [31]; REALM is a reading recognition test that takes approximately 2–3 min to complete [32]; and, finally, the NVS is a 6-item functional health literacy instrument that assesses reading comprehension and numeracy [33]. 

The main objective of this study was to adapt the NVS instrument to the adolescent Portuguese population (NVS-PTeen) and examine its psychometric properties. The NVS instrument was selected because it is a brief, easy-to-administer scale [33,34,35] and has revealed adequate psychometric properties for assessing functional health literacy, including good internal consistency, for adult populations from different cultures, as well as for some adolescent populations (see Table S1, in Supplementary Material, for an extensive list of studies presenting the psychometric properties of the NVS). The original instrument consists of a food nutrition label with six associated questions scored on a dichotomous scale [33]. Although it has been previously adapted for the Portuguese adult population [36,37], its psychometric properties have not been evaluated yet for the adolescent Portuguese population (12 to 17 years old). As such, this is a pioneering, relevant, and timely contribution to the assessment of functional health literacy among Portuguese adolescents.

## 2. Materials and Methods 

### 2.1. Study Design and Setting

A cross-sectional observational study was conducted to assess the internal reliability, as well as the construct and convergent validity, of the NVS-PTeen. Regarding temporal reliability, data collection took place at two different moments, with a 3-month interval. This rather long time interval was set up for minimizing the potential learning effect after applying the instrument to the baseline. 

Self-administered pencil-and-paper questionnaires of the NVS-PTeen were applied in a school setting by a trained researcher. At the beginning of selected lectures, students were asked to fill in the questionnaires, i.e., collective application, with no time restriction. Questionnaires were collected by the same researcher immediately after completion. Due to the collective application of the instrument, the time required for the adolescents to complete the NVS-PTeen was not assessed. 

### 2.2. Sampling and Participants

To evaluate the psychometric properties of the NVS-PTeen, adolescents aged 12 to 17 years and enrolled in grades 8 to 12 in a Portuguese public school were invited to participate in this study (census approach), in a total of 16 classes. Exclusion criteria were (a) being Portuguese native speakers and/or (b) having special educational needs (e.g., due to cognitive impairment). 

There are no clear guidelines about the minimum sample size that is required for assessing psychometric properties of psychosocial scales. Anyway, taking into account the type of statistical analyses considered for this psychometric approach (Spearman’s rank and simple logistic regression, intra-class correlations, exploratory factorial analysis, and item response theory (IRT); further details in the Statistical Analysis section below), a minimum sample size of 250 adolescents was defined. This sample size also corresponds to the median sample size found in a systematic literature review for determining the sample size for validating patient-reported outcome measures [38].

A sub-sample of students participated in the test-retest component of the study. The minimum sample size was settled at 100 students (attending to the nature of the statistical test, without stratification for test-retest analysis). The selection of these participants was done through a random sampling of the classrooms, one class per grade. All students from each randomly selected class were invited to participate in the retest assessment. 

### 2.3. Instruments of Data Collection

Table S1 in Supplementary Material provides the psychometric properties of various adapted and validated versions of the NVS questionnaire for use in different languages and countries. Contents of the NVS-PTeen and its scoring system are available in Table S2 (Supplementary Material). 

The original version of the NVS instrument has been already cross-culturally adapted and validated for the Portuguese adult population [37,39,40], revealing adequate psychometric properties. The authors of the two already mentioned Portuguese versions of the NVS [37,40] followed the standard method for cross-cultural adaptation of instruments by the Scientific Advisory Committee of the Medical Outcomes Trust for linguistic adaptation [41]. In both cases, the authors assumed that cultural issues regarding food labels (typically available on the back of food packages) are not substantially different between the U.S. and Portugal, therefore ensuring conceptually equivalent versions. In the case of the NVS-PTeen, the Portuguese linguistic and cultural adaptation, directly from the original English version, was initially performed for the adult version (same instrument used by Paiva et al. [37]), as follows: (a) two bilingual native Portuguese speakers independently translated the original version of the NVS from English to Portuguese, (b) these two translated versions were merged into a single consensus Portuguese version, (c) the Portuguese consensus version was then back-translated by two bilingual native English speakers, and (d) the two independent English versions were merged into a single consensus version, which was compared to the original NVS instrument. The research team agreed that the two versions did not differ in any relevant way. The NVS-PTeen mainly differs from the adult version by adopting the second-person singular, thus promoting a rather colloquial style. A pre-test was conducted involving three women and three men of different ages (age range: 20–65 years) and with different levels of education (high school and university education).

In addition to the NVS-PTeen, adolescents were asked to provide sociodemographic information, i.e., sex, age, and school year, as well as their final classification results for mathematics and Portuguese disciplines from the previous year. Each questionnaire form included a pre-stamped random individual code, which guaranteed respondents’ anonymity, while ensuring the longitudinal component of the project (only for the test-retest sub-sample of students). 

### 2.4. Statistical Analysis

Descriptive statistics (i.e., mean, median, standard deviation, and frequency) were calculated for sociodemographic indicators (sex, age, and education level). Data normality was assessed using the Kolmogorov–Smirnov non-parametric test (Lilliefors corrected K-S test), complemented with analyses of kurtosis and skewness of the distributions. 

Since the dataset of the total NVS-PTeen score was not normally distributed, comparisons between sexes and among educational levels were performed using Mann–Whitney U and Kruskal–Wallis tests, respectively. The total score for the NVS-PTeen was recoded according to the cutoff points proposed by the authors of the original American English version of the instrument [33]: likelihood of inadequate health literacy (0 to 1 correct answers), limited health literacy (2 to 3 correct answers), and adequate health literacy (4 to 6 correct answers). The percentage of correct/non-correct answers to the NVS-PTeen items and health literacy levels were compared between sexes and among educational levels using chi-square tests. 

Psychometric properties of the NVS-PTeen were evaluated using two different approaches, reliability and validity properties (classic psychometry) and item response theory (IRT). NVS-PTeen reliability was assessed by calculating its internal consistency reliability and its reproducibility (temporal/test-retest reliability). Due to the dichotomous nature of NVS-PTeen items, the internal consistency reliability of this instrument was measured through the Kuder–Richardson 20 (KR20) coefficient [42]. Spearman’s rank correlation and pairwise odds ratios were used for assessing inter-item and item-total associations. A reliability coefficient of 0.70 and a corrected item-total subscale correlation of 0.30 or higher were considered good cutoffs for internal reliability [43]. Test-retest reliability was conducted to assess reproducibility of the NVS-PTeen instrument; as such, the two-way mixed, single-measure intraclass correlation coefficient (ICC) was used.

Concerning validity of the NVS-PTeen, convergent validity and construct validity were assessed. Convergent validity was studied using bivariate correlation analysis (Spearman’s *r* correlation coefficient) between its global score and five theoretically related variables: age, school years, previous-year final classifications for Portuguese and mathematics, and the average final classification of Portuguese and mathematics. Construct validity was studied through exploratory factorial analysis (EFA) with direct oblimin rotation, following the same procedure as used in previous studies of the NVS (e.g., [24,44]). The Kaiser–Meyer–Olkin (KMO) test and Bartlett’s test of sphericity were performed to determine assumptions of EFA and sampling adequacy for principal component analysis. The correlation matrix of all six items and mean inter-item correlation were verified to evaluate the strength of association between the items. An eigenvalue higher than 1 and a screen plot were used to determine the number of factors. After oblimin rotation, items with a factor loading of 0.40 or greater were considered adequate for measuring a factor.

Finally, IRT was used for estimating item difficulty, discrimination, and fit. Item difficulty refers to the level of health literacy required to meet at least 50% chance of correctly answering an item; item discrimination refers to the capacity of an item to differentiate students with high health literacy from students with low health literacy (items with discrimination values below 1 indicate less discriminating efficacy); and item fit refers to the degree to which observed responses to an item correspond to expected responses, given the difficulty of the item and the respondent’s level of health literacy. Values above 0.8 indicate an adequate item fit [45].

Statistical analyses were performed with IBM Statistical Package for the Social Sciences (SPSS), version 24.0, and with jMetrik, version 4.0.6, for IRT analysis. Statistical significance was set to α = 0.05.

### 2.5. Ethical Considerations

Authorization for adapting and validating the NVS instrument for the Portuguese population was granted from Pfizer, Inc., the company that holds its copyright, and approved by the Ethical Committees of the Universidade do Porto and Centro Académico de Medicina de Lisboa. 

The assessment of the psychometric properties of the Portuguese version of the NVS-PTeen followed the guidelines laid down in the Declaration of Helsinki, amended in Fortaleza [46]. Data collection was approved by the direction board of the school where data collection took place. Prior to study enrollment, the adolescents were informed of the study objectives, of its disassociation from the curricular activities, and that their participation was voluntary, with no impact on their academic activities and/or results. Furthermore, they were explained that the filling in of the questionnaires was part of a research study, not a school test/exam and also that teachers would not have access to the results of the NVS-PTeen. Only the adolescents whose parents signed a consent form (with detailed information about the goals and tasks of the project) and who confirmed their willingness to participate were involved in the study. Anonymity was not possible for the students enrolled in the longitudinal component of the study. However, it was explained that only members of the research team would have access to the data collected, which would be kept confidential, and that no personal data allowing their identification (i.e., name) would be recorded in the main database (a random code was attributed to each student for test-retest matching).

## 3. Results

### 3.1. Sample Characterization

Overall, 386 students (48.8% female) from 16 different classes participated in the study (Table 1). Regarding upper secondary education (grades 10 to 12), 28 students were not enrolled in mathematics courses and, therefore, only classification marks from Portuguese classes were used. Participants were aged, on average, 14.4 (SD = 1.4) years; no statistically significant differences between boys and girls were found (*p* = 0.79). In addition, no significant association was found between being a male or a female and the grade in which the students were enrolled; about 60% of the sample was enrolled in lower secondary education (grades 8 to 9; Table 1). About 10% of the students had negative scores in Portuguese in the previous academic year, whereas 20% of them scored negative in mathematics (normal distributions for students in grades 8 to 9; non-normal, left-skewed distribution for students in grades 10 to 12; data not provided).

### 3.2. Functional Health Literacy among Adolescents

Girls failed slightly more NVS-PTeen questions than boys, except for items 5 and 6 (Table 2). However, statistically significant differences between boys and girls were only found for item 1 (*p* = 0.014). The prevalence of adequate health literacy was high for both sexes (80.6% for girls and 86.7% for boys), with no statistically significant differences (*p* = 0.26). In addition, 44.0% of the total sample answered the six questions of the NVS-PTeen correctly, thus obtaining the maximum score (score = 6; 45.0% for girls, 43.1% for boys).

The educational level (i.e., number of school years) was significantly associated with all NVS-PTeen items (*p* < 0.05), except for items 1 and 5 (Table 2). The prevalence of compromised health literacy was higher among students in grade 8 (4.8% of the students had inadequate health literacy and 23.8% had limited health literacy) and grade 9 (3.8% of the students had inadequate health literacy and 16.3% had limited health literacy) than students in grades 10 to 12 (*p* < 0.001). Indeed, adequate health literacy was detected among more than 90% of the students in grades 10 to 12. 

### 3.3. Internal Consistency and Test-Retest Reliability

The overall internal consistency reliability of the self-administered NVS-PTeen was KR20 = 0.61 (95% CI = 0.54–0.66).

The inter-item Spearman’s rank correlation coefficients and odds ratios are provided in Table 3. All inter-item correlation and odds ratio were statistically significant, except for the pairs of items 2 and 3, and 2 and 6. The highest inter-item Spearman’s rank correlation coefficients and odds ratios were obtained for the pair of items 5 and 6 (*r* = 0.59). Regarding the item-total correlation, which is an item discrimination indicator, Spearman’s rank correlation coefficients ranged from *r* = 0.49 (item 3) to *r* = 0.67 (item 4) (Table 3). 

The test-retest reliability was acceptable (ICC = 0.605; 95% CI = 0.54–0.66). The majority of deviations were toward improved health literacy from the first to the second observation. Item 2 had low consistency (Table 4).

### 3.4. Convergent Validity of the NVS-PTeen

As presented in Table 5, a weak correlation was found between age and the NVS-PTeen total score. The educational level, given as the number of school years the student was enrolled for at the time of data collection, and final classifications of Portuguese and mathematics from the previous academic year were moderately correlated with the NVS-PTeen final score. 

### 3.5. Construct Validity-Dimensionality

**Figure 1:**
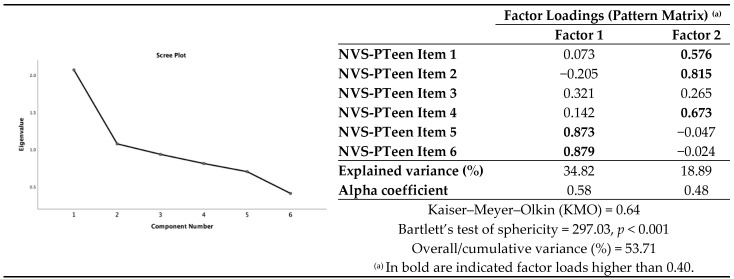
Exploratory factor analysis after direct oblimin rotation (with the screen test plot of eigenvalues) for the NVS-PTeen (*N* = 386).

The Kaiser–Meyer–Olkin (KMO) test suggested an adequate fit (KMO = 0.64) of the dataset for factorial analysis. A similar result was obtained after Barlett’s test of sphericity, which indicated that the correlation matrix was significantly different from zero (*p* < 0.001) and, thus, suitable for factorial analysis. Two factors were obtained by means of factorial analysis with direct oblimin rotation, eigenvalues above 1 and factor loading above 0.4. The eigenvalues for these two factors were 2.09 and 1.13, with 34.82% and 18.89% of the explained variance, respectively. The two factors, comprising the six items, explained 53.71% of the total variance (Figure 1). Factor 1 was associated with reading-related literacy, while factor 2 was associated with numeric skills. Item 3 revealed to be weakly associated with both factors.

### 3.6. Item Response Theory (IRT): Item Difficulty, Discrimination, and Fit

**Figure 2:**
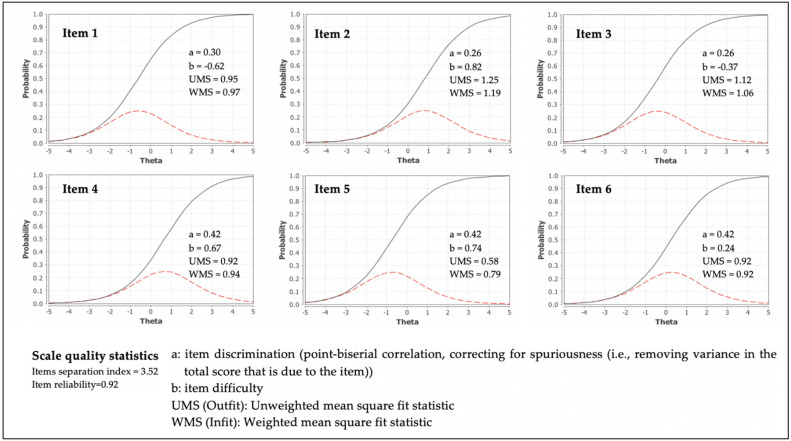
NVS-PTeen items: characteristic and information curves (*N* = 386).

After adjusting for spuriousness, i.e., after removing the variance attributable to the NVS-PTeen total score due to the item-specific variance, IRT curves (Figure 2) revealed that items 4, 5, and 6 were the most discriminative ones (a = 0.42), whereas items 2 and 3 were the least discriminative ones (a = 0.26). Concerning item difficulty, items 3 and 6 scored as the easiest ones (b = −0.37 and b = 0.24, respectively), whereas item 2 was the most difficult one (b = 0.82). Finally, the fit between observed and expected responses was adequate for all items (UMS, unweighted mean squares, and WMS, weighted mean squares, values ranged between 0.8 and 1.2), except for item 5.

## 4. Discussion

In this study, the psychometric properties of a self-administered version of the Newest Vital Sign for adolescents, the NVS-PTeen were assessed by combining classic and modern (IRT) psychometric tools. Although there are a few published studies that assess health literacy in Portuguese samples [37,39,40], none of them specifically targets adolescents. The main findings of this observational cross-sectional study (with a longitudinal component) are as follows: (a) functional health literacy levels for 83.4% of the participants were adequate; (b) upper secondary students had higher functional health literacy levels than lower secondary education students; (c) overall, the NVS-PTeen had acceptable psychometric properties measured using the classical methods (i.e., reliability and validity); and (d) the analysis of IRT curves allowed the identification of the most discriminative and easiest items in the instrument and revealed a good fit between observed and expected answers.

### 4.1. Prevalence of Adequate Health Literacy in the Adolescent Sample under Study

Health literacy assumes an indisputable role in supporting adequate and effective health decisions. Indeed, available evidence supports the association between inadequate health literacy, decreased health outcomes, increased healthcare use, and increased health expenditure (e.g., [47,48,49]). Since health and health behaviors during childhood and adolescence are strongly associated with health outcomes during adulthood [23,24,25], adequate health literacy assumes particular relevance during early life as a health promotion strategy across the life span. However, health literacy data during childhood and adolescence is scarce [12,15], which might be due to a combination of factors, namely no consensus regarding the definition of the health literacy construct [50] and a lack of adequate tools for measuring this indicator at this life stage [13,29]. In addition, there has been some debate on whether to measure health literacy among children. Indeed, as defended by Weiss, “Why would we expect children, particularly elementary school children (some of whom, such as the 7-year-olds, are still learning to read) to be able to interpret the complexities of a nutrition label, something that even many adults cannot do” [51] (p. e19). This argument is less tenable for adolescents because analytical thought, namely regarding text interpretation and numerical/arithmetic capabilities, is mostly developed at these ages [52]. Therefore, it is highly relevant to evaluate how school-related achievements are effectively contributing to the improvement of (applied) functional health literacy. In the particular case of Portugal, the few studies available have mainly focused on measuring youth mental (content-specific) health literacy (e.g., [53]), whereas data on functional health literacy for adolescents aged below 18 years have been generally disregarded.

The prevalence of adequate functional health literacy, measured with the NVS, among Portuguese adolescents participating in this study was higher than among US adolescents aged 12–19 years (51%) [54]. The lack of prevalence measures of functional health literacy for adolescents, with a particular focus on European adolescents, measured with the NVS instrument or even with other measuring tools, precludes the comparison of our data with results from elsewhere. As such, the results from this study are coarsely placed in a wider context. The prevalence of adequate functional health literacy reported here contrasts with the one by Paiva et al. [37] for a representative sample of the adult Portuguese population (aged 16 to 79 years), measured using the same NVS instrument. Paiva et al. [37] reported considerably lower levels of adequate health literacy (27.1%) than the ones reported here (83.4%). Results from the European Health Literacy Survey (HLS-EU), an eight-country survey of health literacy (European citizens aged ≥15 years), revealed highly variable levels of adequate health literacy, including the NVS instrument as a component of the assessment [8]. These ranged from 36.9%, in Spain, to 76.3%, in the Netherlands; in Portugal, the same HLS-EU methodological approach revealed high levels of inadequate health literacy, as previously mentioned in the Introduction section [9,10]. Multiple factors might explain the differences in the average prevalence values reported across studies, including sample heterogeneity in terms of sociodemographic characteristics. Indeed, individuals with lower socioeconomic status, lower educational level, and higher age are more vulnerable to low functional health literacy than their counterparts [1,8].

The educational level plays a key role in functional health literacy [1,8]. Paiva et al. [37] detected a significant association between educational level and functional health literacy in the adult Portuguese population. In their study, individuals who completed a university degree provided more correct answers than those with a lower education level. For example, 55.5% of those with a university degree had adequate literacy versus 25.9% of those who completed lower secondary education [37]. In the particular case of our study, the overall prevalence of adequate health literacy among adolescents was much higher. The most plausible explanation for such higher prevalence of functional health literacy when compared to results from other studies has to do with sampling—only one school was involved in this study. Despite the heterogeneity in terms of social and economic family backgrounds of the students enrolled in this school, teaching methods are somehow homogenous across classes (e.g., the same teacher teaches more than one class per grade and even classes from different grades) and the Portuguese and mathematics classification marks from the previous year were generally good. It is worth mentioning that in the year before data was collected, this school ranked 154 position (out of 593) in the national secondary education schools ranking [55]. Thus, the prevalence values of functional health literacy provided here cannot be generalized for the adolescent population in Portugal. 

### 4.2. Psychometric Properties of the NVS-PTeen

The main endeavor of this study was to investigate the psychometric properties of the NVS instrument for the Portuguese population aged 12 to 17 years. The combination of classical and modern methods for assessing the psychometric properties of the NVS-PTeen instrument used here allowed a comprehensive understanding of the global instrument, but also of each item separately. Several previous studies that adapted, validated, and investigated the psychometric properties of the NVS instrument used a face-to-face (hetero-)administered questionnaire (see Table S1 in Supplementary Material for an extensive list of these studies). In this study and a few others [40,56,57,58,59], the questionnaire was self-administrated with a potential reduced burden of administration compared to its hetero-administration [60]. Indeed, available evidence indicates that self-administered versions of the NVS can take up to 6 min to complete [40,59], whereas hetero-administered versions of the questionnaire can take up to 8 min to complete [33]. Unfortunately, due to the collective self-administration of the NVS instrument in the school-class setting employed in this study, no data on the completion time were gathered.

The internal consistency of the NVS-PTeen was acceptable as estimated with KR20 (KR20 = 0.61), a special case of Cronbach’s α for dichotomous variables. This finding contrasts with good internal consistency reported for the original NVS instrument (Cronbach’s α = 0.76) by Weiss et al. [33], as well as for the adult Portuguese version of this instrument (Cronbach’s α = 0.85) by Paiva et al. [37] and others who assessed functional health literacy among adolescents [54,56,61,62]. Nevertheless, the coefficient α (i.e., Cronbach’s α and KR20) is sensitive to scale length, and it tends to be lower for shorter instruments [63]. As such, internal consistency of the NVS-PTeen is satisfactory, given that this is a short-length scale with six dichotomous (correct/incorrect) scoring-format items. In addition, as it is widely acknowledged, coefficient α should not be used as the only measure of internal consistency of an instrument; inter-item associations are also useful for a comprehensive assessment of its internal consistency [64]. In this study, inter-item correlation and odds ratio values were satisfactory (broadly within the interval 0.20–0.40, with a few exceptions) and similar to the ones previously obtained by Martins and Andrade [40] for a Portuguese clinical (adult) sample. Reproducibility of the instrument, as assessed by test-retest, was also acceptable (ICC = 0.605); similar findings were previously reported by Cruvinel et al. [65] (ICC = 0.57), Zotti et al. [66] (Spearman’s *r* = 0.65), and Kogure et al. [57] (Pearson’s *r* = 0.82).

Moderate convergent validity between the NVS-PTeen total score and education-related indicators (i.e., educational level, previous-year classifications of Portuguese and mathematics) was detected. This is particularly informative because the NVS-PTeen assesses functional health literacy, mainly requiring reading and numeracy skills [33]. The low correlation coefficient obtained between the NVS-PTeen total score and age adds support to this claim and suggests that the scoring of this instrument is not affected by potential developmental bias. Concerning construct validity, two factors, each reflecting a particular aspect of functional literacy—reading and numeracy skills—were identified and collectively explained 53.71% of the variance in the six-item questionnaire, a slightly lower value than the one found by Martins and Andrade [40] for their Portuguese version of the NVS instrument (60.97%). Finally, the overall analysis of the psychometric properties of the NVS-PTeen instrument using the classical test theory generally agreed with the findings from the analysis of the IRT curves.

A considerable ceiling effect was detected in this study: 44.0% of the respondents scored the maximum value for the NVS-PTeen. The same effect, although less pronounced, was also reported for versions of the NVS instrument administered to community dwellers in the UK [34] and in the Netherlands [67], as well as for English children [56] and US adolescents [54]. Interestingly, the opposite effect (i.e., floor effect) was reported by Fransen et al. [68] and Kogure et al. [57] for clinical samples of adults and also for Portuguese older community dwellers [39]. Scoring near the possible upper or lower limit of the NVS instrument seems to be a major limitation precluding the discrimination among individuals on the top or bottom ends of the scale. In the particular case of this study, the ceiling effect may be due to sampling, which involved students from only one school. This methodological approach guaranteed control over data collection, including data quality, but did not mirror the Portuguese context at all; this school was well ranked in terms of Portuguese and mathematics final scores, as previously addressed [55], and this may have had some effect on the results. Nevertheless, the main purpose of this study was to adapt and validate the NVS instrument for the adolescent Portuguese population and not to investigate Portuguese adolescents’ functional health literacy. As such, it can be concluded that the NVS-PTeen is an adequate, low-burden screening tool for functional health literacy in a very specific, relevant, and less studied age group.

### 4.3. Strengths and Limitations

For a comprehensive analysis of the results of this study, its strengths and limitations should be acknowledged. Concerning the strengths, this is the first adaptation and psychometric study of the NVS for the adolescent Portuguese population, in a self-administered format. Previous adaptation and validation studies of this instrument for the Portuguese population concerned only adult community dwellers [37,39,40]. Second, contrary to previous studies (e.g., [39,40,69]), a gender-balanced sample was enrolled; the sample size was also highly satisfactory (*N* = 386) [70]. A major limitation of this study was sampling. As mentioned above, all sampled students came from only one school, which precludes the generalization of the results on functional health literacy to the adolescent Portuguese population. Moreover, this non-probabilistic approach much probably contributed to the marked ceiling effect observed, as discussed above. Another limitation was with regard to the fact that the NVS-PTeen is, in essence, identical to the adult Portuguese version (only differing from the adult version by adopting the second-person singular, a more colloquial and adequate tone when addressing adolescents). This might be a limitation because no additional adolescent-cultural-adapted efforts have been made, specifically for the Portuguese population. Additional research, using cognitive interview processes, would be adequate to better understand if this instrument would benefit from changes, both in the food label that is presented to respondents and/or regarding the writing of the questions. Nevertheless, the NVS-PTeen revealed acceptable psychometric properties, which indicates that this instrument can confidently be administered to a larger and more heterogeneous sample of Portuguese adolescents.

## 5. Conclusions

Serious health issues in adulthood result from multiple risk behaviors established during adolescence, including alcohol and other substances abuse, sexual risk-taking behaviors, tobacco use, unhealthy eating habits, and little or no physical activity, among others [71]. Under this context, adolescents attaining higher levels of functional health literacy will potentially have improved health outcomes during the transition to adulthood and as adults [12,14]. Nevertheless, literacy among adolescents has been reported to differ between socioeconomic status and ethnicity (none of these predictors were formally assessed here) [12]. As such, actions addressing health literacy asymmetries between specific groups of adolescents assume a relevant role as cost-effective health promotion strategies in the life span and also as avenues toward reduction in health inequities. 

Schools have been increasingly recognized as key settings for achieving health literacy (e.g., [26,27,28]). This is because the school environment potentially provides the resources and supportive environment that facilitate behavioral change and skill improvement, thus contributing to the prevention of multiple risk behaviors during adolescence and adulthood (e.g., [72]). The significant correlations reported here between NVS-PTeen scores and mathematics and Portuguese final classifications put into evidence the role of schools in active health promotion rather than simply providing the students with specific disease- or health-related information. Learning skills and analytical and objective thought are main developmental tasks that should be promoted at school as prerequisites for achieving functional health literacy. As argued by Winkelman et al. [73], there are four steps needed for improving health literacy at schools: (a) curricula covering health education topics, (b) funding, (c) partnerships between the healthcare and education sectors, and (d) the incorporation of health-literacy-screening systems, namely through the use of easy-to-administer, valid, and reliable tools. Results herein provide some support for the use of the NVS-PTeen to assess functional health literacy among Portuguese adolescents. The usage of adequate instruments to assess functional health literacy has the potential to promote valuable insight for both public health and education institutions concerning the relationship between health literacy, health behavior, and health outcomes in adolescents (and adulthood). We are confident that the NVS-PTeen is a useful health education monitoring tool if used regularly in the school context (e.g., at the end of each school cycle) in order to evaluate how official educational curricula may be translated into functional health literacy. Because health literacy is a pivotal variable for public health promotion through health education initiatives [4], this instrument may, therefore, constitute a relevant tool for assessing public health promotion among adolescents.

## Figures and Tables

**Table 1:** Sample characterization: age and educational level of the participants by sex.

		Female (*n* = 191)	Male (*n* = 195)	Total (*N* = 386)	*p*-Value ^1^
Age (years)	12	14 (7.3%)	14 (7.2%)	28 (7.3%)	0.672
	13	49 (25.7%)	45 (23.1%)	94 (24.4%)	
	14	39 (20.4%)	54 (27.7%)	93 (24.1%)	
	15	35 (18.3%)	30 (15.4%)	65 (16.8%)	
	16	33 (17.3%)	34 (17.4%)	67 (17.4%)	
	17	21 (11.0%)	18 (9.2%)	39 (10.1%)	
	Mean (±SD)	14.46 (1.49)	14.41 (1.43)	14.43 (1.46)	0.791
	Median	14.00	14.00	14.00	
Educational level	Grade 8	66 (34.6%)	60 (30.8%)	126 (32.6%)	
	Grade 9	43 (22.5%)	61 (31.3%)	104 (26.9%)	
	Grade 10	29 (15.2%)	21 (10.8%)	50 (13.0%)	0.321
	Grade 11	35 (18.3%)	36 (18.5%)	71 (18.4%)	
	Grade 12	18 (9.4%)	17 (8.7%)	35 (9.1%)	

^1^*p*-Value calculated using the chi-square test for categorical variables and the Mann–Whitney *U* test for continuous variables.

**Table 2:** The Newest Vital Sign for the Portuguese adolescents’ population (NVS-PTeen) score comparison for (a) sex and (b) educational level.

		NVS-PTeen Items (I)						NVS Total Score (Range: 0–6; Mean ± SD)	Health Literacy Level		
		I1 (%)	I2 (%)	I3 (%)	I4 (%)	I5 (%)	I6 (%)		Inadequate (Score 0–1; %)	Limited (Score 2–3; %)	Adequate (Score 4–6; %)
Sex	Female (*n* = 191)	84.3	71.2	83.2	71.2	90.6	79.1	4.72 ± 1.41	3.7	15.7	80.6
	Male (*n* = 195)	92.3	71.3	88.7	75.4	88.2	79.0	4.86 ± 1.27	2.1	11.3	86.7
	*p*-value ^1^	**0.014**	0.987	0.121	0.353	0.450	0.984	0.526	0.259		
Educational level	Grade 8 (*n* = 126)	84.1	61.1	86.5	57.1	84.9	63.5	4.27 ± 1.47	4.8	23.8	72.2
	Grade 9 (*n* = 104)	85.6	73.1	76.9	64.4	86.5	78.8	4.50 ± 1.31	3.8	16.3	79.8
	Grade 10 (*n* = 50)	94.0	94.0	96.0	96.0	96.0	92.0	5.68 ± 0.89	2.0	2.0	96.0
	Grade 11 (*n* = 71)	90.1	73.2	88.7	88.7	95.8	91.0	5.28 ± 1.03	0.0	5.6	94.4
	Grade 12 (*n* = 35)	100.0	65.7	91.4	97.1	91.4	94.3	5.29 ± 0.83	0.0	2.9	97.1
	*p*-value ^2^	0.053	**0.001**	**0.013**	**<0.001**	0.060	**<0.001**	**<0.001**		**<0.001**	
Total (*N* = 386)		88.3	71.2	86.0	73.3	89.4	79.0	4.79 ± 1.34	2.8	13.5	83.4

^1^*p*-Value calculated using the chi-square test for percentages and the Mann–Whitney *U* test for continuous variables; ^2^ *p*-value calculated with the chi-square test for percentages and the Kruskal–Wallis test for continuous variables. Statistically significant values are in bold.

**Table 3:** Inter-item and item-total NVS-PTeen scores (Spearman’s rank correlation coefficients matrix and inter-item pairwise odds ratio (OR) with 95% confidence intervals (95% CI); *N* = 386).

	Item 2		Item 3		Item 4		Item 5		Item 6		NVS-PTeen Total Score
	rho	OR (95% CI)	rho	OR (95% CI)	rho	OR (95% CI)	rho	OR (95% CI)	rho	OR (95% CI)	rho
**Item 1:** *If you eat the whole container of ice cream, how many calories are you going to consume?*	0.197 **	3.33 (1.77–6.29)	0.179 **	3.40 (1.67–6.93)	0.201 **	3.41 (1.81–6.44)	0.163 **	3.35 (1.54–7.29)	0.170 **	2.94 (1.53–5.67)	0.445 **
**Item 2:** *If you could eat 60 g of carbohydrates, what quantity of ice cream would you be able to eat?*			0.057	1.42 (0.77–2.60)	0.315 **	4.37 (2.69–7.09)	0.115 *	2.12 (1.10–4.11)	0.094	1.63 (0.97–2.73)	0.596 **
**Item 3:** *Your doctor has advised you to reduce the amount of saturated fat in your diet. You generally eat 42 g of saturated fat per day, which includes one portion of ice cream. If you were to stop eating ice cream, how many grams of saturated fat would you be consuming per day?*					0.213 **	3.37 (1.86–6.09)	0.176 **	3.44 (1.65–7.17)	0.196 *	3.18 (1.73–5.86)	0.447 **
**Item 4:** *If you generally eat 2500 calories per day, what percentage of the daily value of calories would you be consuming if you ate one portion of ice cream?*							0.210 **	3.77 (1.95–7.32)	0.264 **	3.75 (2.24–6.28)	0.662 **
**Item 5:** *Is it safe for you to eat this ice cream?*									0.586 *	63.28 (21.51–186.15)	0.498 **
**Item 6** (asked if the participant answered no to item 5): *Why not?*											0.599 **

* *p* < 0.05; ** *p* < 0.01.

**Table 4:** Internal consistency (coefficient α) and reproducibility of the NVS-PTeen given as temporal reliability.

	Coefficient α If Item Deleted (*N* = 386)	Item Difficulty: % of Students Answering the Item Correctly (*N* = 386)	Temporal Reliability	
			% of Test-Retest Accuracy (*n* = 127)	% of Test-Retest Score Improvement (*n* = 127)
Item 1	0.58	88.3%	96.9%	0.8%
Item 2	0.60	71.2%	66.1%	27.6%
Item 3	0.59	86.0%	78.7%	7.1%
Item 4	0.52	73.3%	81.9%	6.3%
Item 5	0.53	89.4%	92.9%	3.1%
Item 6	0.53	79.0%	86.6%	10.2%

**Table 5:** Convergent validity of the NVS-PTeen assessed as the correlation between theoretically related variables and the NVS-PTeen total score.

	Pearson’s *r*
Age (*N* = 386)	0.19 *
Educational level (*N* = 386)	0.31 **
Final classification: Portuguese ^1^ (*N* = 386)	0.28 **
Final classification: mathematics ^1^ (*N* = 358)	0.31 **
Average final classification: Portuguese and mathematics ^1^ (*N* = 358)	0.39 **

^1^ Final classifications from the previous academic year; * *p* < 0.05; ** *p* < 0.001.

## Data Availability

The data presented in this study are available on request from the corresponding author.

## Supplementary Materials

The following are available online at https://www.mdpi.com/2072-6643/13/3/790/s1, Table S1: Selected published psychometric studies of the Newest Vital Sign. Table S2: Newest Vital Sign | Portuguese adolescent population version (NVS-PTeen).
