# Supplementary material for: Functional Health Literacy: Psychometric Properties of the Newest Vital Sign for Portuguese Adolescents (NVS-PTeen)
Source: Nutrients. 2021 Feb 27;13(3):790. doi: 10.3390/nu13030790 (PMC7997379; doi:10.3390/nu13030790)
Supplement: Supplementary file 1 [file nutrients-13-00790-s001.pdf]

Functional health literacy: psychometric properties of the NVS for the adolescent Portuguese population

Osvaldo Santos [1,2,3]*, Miodraga Stefanovska-Petkovska [1], Ana Virgolino [1,3], Ana Cristina Miranda [1], Joana Costa [1], Elisabete Fernandes [3], Susana Cardoso [4,5] and António Vaz Carneiro [1,3,6,7]

Supplementary materials

Table S1. Selected published psychometric studies of the Newest Vital Sign.

**Reference**	**Format of administration**	**Country**	**Language**	**Age of the respondents**	**Population**	**Reliability (Cronbach α)**	**Convergent validity** correlation coefficient (comparator)
Al-Jumaili et al., 2015 [1]	Self-administration	Iraq	Arabic	≥ 15	Community	0.69	0.513 (S-TOFHLA)<br>0.089 (SILS)
Berens et al., 2016 [2]	Hetero-administration	Germany	German	≥ 15	Community	0.73	Not reported
Brangan et al., 2018 [3]	Hetero-administration	Croatia	Croatian	18-84	Clinical	Not reported	Not reported
Caldwell et al., 2018 [4]	Self-administration	USA	English	10-19	Clinical	0.63	0.38 (REALM-Teen)
Carpenter et al., 2015 [5]	Hetero-administration	USA	English	≥ 18	Clinical	0.78	0.602 (S-TOFHLA)
Cruvinel et al., 2018 [6]	Self-administration	Brazil	Portuguese	18-80	Clinical	0.79	0.60 (BREALD-30)<br>0.54 (NFLI)
Driessnack et al., 2014 [7]	Self-administration	United kingdom	English	07-12<br>⩾ 18	Community	0.71<br>0.79	0.36 (HLE, Children)<br>0.44 (HLE, Parents)
Dunn-Navarra et al., 2012 [8]	Hetero-administration	USA	English and Spanish	19-53	Community	Not reported	0.51 (S-TOFHLA)
Fransen et al., 2011 [9]	Hetero-administration	Netherlands	Dutch (based on the original version of the NVS)	≥ 18	Clinical	0.67	0.32 (REALM)<br>0.22 (SBSQ)<br>0.18 (FCCHL)
Fransen et al., 2014 [10]	Hetero-administration	Netherlands	Dutch (country-adapted version of the food label used in NVS)	18-75	Community	0.76	0.20 HLS-EU-Q16<br>0.53 (SAHL-D)<br>0.49 (Prose literacy)
Hoffman et al., 2013 [11]	Self-administration	USA	English	14-19	Community	0.67	0.49 (TerraNova)
Hoffman et al., 2015 [12]	Hetero-administration	Guatemala	Spanish	10-16	Community	Not reported	Not reported; results from RASCH analysis reported instead
Howe et al., 2018 [13]	Hetero-administration	USA	English	07-12	Community	0.71	0.20 (child age)<br>0.24 (school grade)

Reference	Format of administration	Country	Language	Age of the respondents	Population	Reliability (Cronbach α)	**Convergent validity** correlation coefficient (comparator)
Huang et al., 2018 [14]	Hetero-administration	USA	English	≥ 20	Clinical	0.80	Not reported
Kirk et al., 2011 [15]	Hetero-administration	USA	Not report	≥ 60	Community	Not reported	0.54 (S-TOFHLA)
Kogure et al., 2014 [16]	Self-administration	Japan	Japanese	≥ 18	Clinical	0.72	0.43 (HLQ total)<br>0.39 (HLQ functional)<br>0.72 (CGI)
Kordovski et al., 2017 [17]	Self-administration	USA	English	51.6 (mean)	Clinical and community	0.70	0.47 (REALM)<br>0.39 (SILS)
Manganello et al., 2015 [18]	Hetero-administration	USA	English	10-19	Clinical and community	0.76	-0.26 (HAS-A)
Mansfield et al., 2018 [19]	Self-administration	Canada	English and French	≥ 18	Community	Not reported	Not reported
Martins and Andrade, 2014 [20]	Self-administration	Portugal	Portuguese	18-94	Community and clinical	0.67	0.29 (SES)
Miser et al., 2013 [21]	Hetero-administration	USA	English	≥ 18	Clinical	0.80	0.62 (S-TOFHLA)<br>0.39 (SKILLD)
Morrison et al., 2014 [22]	Hetero-administration	USA	English and Spanish	18-69	Clinical	Not reported	0.32 (S-TOFHLA)
Norrafizah et al., 2016 [23]	Self-administration	Malaysia	Malay	≥ 18	Community	Not reported	Not applicable
Osborn et al., 2007 [24]	Hetero-administration	USA	English	≥ 18	Clinical	0.81	0.41 (REALM)<br>0.61 (S-TOFHLA)
Ozdemir et al., 2010 [25]	Hetero-administration	Turkey	Turkish	≥ 18	Clinical	0.70	0.52 (REALM)
Paiva et al, 2017 [26]	Hetero-administration	Portugal	Portuguese	≥ 18	Community	0.85	Not reported; discriminant validity reported instead
Ramirez-Zohfeld et al., 2015 [27]	Hetero-administration	USA	English and Spanish	50-75	Clinical	Not reported	0.69 (S-TOFHLA)
Rodrigues et al., 2017 [28]	Hetero-administration	Brazil	Portuguese	19-68	Community	0.75	Not reported; exploratory & confirmatory factor analysis reported instead
Rowlands et al., 2013 [29]	Hetero-administration	United Kingdom	English	≥ 18	Community	0.74	0.49 (TOFHLA)
Russell et al., 2019 [30]	Hetero-administration (face-to-face and by phone interview)	USA	English	≥ 18	Clinical	0.76	Not applicable
Tseng et al., 2018 [31]	Self-administration	Taiwan	Traditional Chinese	≥ 30	Clinical	0.70	0.59 (NLS)<br>0.38 (DNKT)<br>0.18 (3BSQ)
Warsh et al., 2014 [32]	Hetero-administration	USA	English	07-17	Clinical	Not reported	0.71 (GSRT)
Weiss et al., 2005 [33]	Hetero-administration	USA	English and Spanish	18-25	Clinical	0.76 (English version)<br>0.69 (Spanish version)	0.59 (TOFHLA)

Reference	Format of administration	Country	Language	Age of the respondents	Population	Reliability (Cronbach α)	Convergent validity correlation coefficient (comparator)
Wolf et al., 2012 [34]	Hetero-administration	USA	English	55-74	Clinical	Not reported	0.62 (TOFHLA)<br>0.47 (REALM)
Xue et al., 2018 [35]	Self-administration combined with hetero-administration	China	Chinese	≥ 18	Community	0.71	0.68 (CCHLQ)
Zotti et al., 2017 [36]	Hetero-administration	Italy	Italian	18-65	Clinical	0.74	0.58 (STOFHLA)<br>Significant for SrRA, although the value was not reported<br>Not reported and non-significant for SILS

TOFHLA, Test of Functional Health Literacy in Adults; REALM, Rapid Estimate of Adult Literacy in Medicine; SBSQ, Set of Brief Screening Questions; FCCHL, Functional Communicative and Critical Health Literacy; HLS-EU-Q16, Health Literacy Survey-Europe; SAHL-D, Short Assessment of Health Literacy for Dutch Patients; HLQ, Health Literacy Questionnaire; CGI-HL, Clinical Global Impression Scale; CGI-HL, Clinical Global Impression Scale of Participants' Comprehensive Health Literacy levels; HLE, Home Literacy Environment single-question screening item; SES, Self-Efficacy Scale; GSRT, Gray Silent Reading Test; HAS-A, Health Literacy Assessment Scale for Adolescents; NLS, Nutrition Label Survey; DNKT, Diabetes Nutrition Knowledge Test; 3BSQ, 3-Brief Screening Questions; BREALD-30, Brazilian version of the Rapid Estimate of Adult Literacy in Dentistry; NFLI, National Functional Literacy Index; CCHLQ, Chinese Citizen Health Literacy Questionnaire; SrRA, Self-rated Reading Ability; SILS, Single Item Literacy Screener; S-TOFHLA, Short Test of Functional Health Literacy in Adults; TerraNova, TerraNova standardized reading assessment.

References (in Table 1)

1. Al-Jumaili, A.A.; Al-Rekabi, M.D.; Sorofman, B. Evaluation of instruments to assess health literacy in Arabic language among Iraqis. *Res. Soc. Adm. Pharm.* **2015**, *11*, 803–813, doi:10.1016/j.sapharm.2015.02.002.
2. Berens, E.M.; Vogt, D.; Messer, M.; Hurrelmann, K.; Schaeffer, D. Health literacy among different age groups in Germany: results of a cross-sectional survey. *BMC Public Health* **2016**, *16*, 1–8, doi:10.1186/s12889-016-3810-6.
3. Brangan, S.; Ivanišić, M.; Rafaj, G.; Rowlands, G. Health literacy of hospital patients using a linguistically validated Croatian version of the Newest Vital Sign screening test (NVS-HR). *PLoS One* **2018**, *13*, e0193079, doi:10.1371/journal.pone.0193079.
4. Caldwell, E.P.; Carter, P.; Becker, H.; Mackert, M. The Use of the Newest Vital Sign Health Literacy Instrument in Adolescents With Sickle Cell Disease. *J. Pediatr. Oncol. Nurs.* **2018**, *35*, 361–367, doi:10.1177/1043454218767875.
5. Carpenter, C.; Kaphingst, K.; Goodman, M.; Lin, M.; Melson, A.; GriffeyRT Feasibility and Diagnostic Accuracy of Brief Health Literacy and Numeracy Screening Instruments in an Urban Emergency Department. *Acad Emerg Med* **2014**, *21*, 1–19, doi:10.1111/acem.12315.
6. Cruvinel, A.F.P.; Méndez, D.A.C.; Chaves, G.C.; Gutierres, E.; Lotto, M.; Oliveira, T.M.; Cruvinel, T. The Brazilian validation of a health literacy instrument: the Newest Vital Sign. *Acta Odontol. Scand.* **2018**, 1–8, doi:10.1080/00016357.2018.1484511.
7. Driessnack, M.; Chung, S.; Perkhounkova, E.; Hein, M. Using the "Newest Vital Sign" to assess health literacy in children. *J. Pediatr. Heal. Care* **2014**, *28*, 165–171, doi:10.1016/j.pedhc.2013.05.005.
8. Dunn-Navarra, A.M.; Stockwell, M.S.; Meyer, D.; Larson, E. Parental health literacy, knowledge and beliefs regarding upper respiratory infections (uri) in an urban latino immigrant population. *J. Urban Heal.* **2012**, *89*, 848–860, doi:10.1007/s11524-012-9692-8.
9. Fransen, M.P.; Van Schaik, T.M.; Twickler, T.B.; Essink-Bot, M.L. Applicability of internationally available health literacy measures in the Netherlands. *J. Health Commun.* **2011**, *16*, 134–149, doi:10.1080/10810730.2011.604383.
10. Fransen, M.P.; Leenaars, K.E.F.; Rowlands, G.; Weiss, B.D.; Maat, H.P.; Essink-Bot, M.L. International

application of health literacy measures: Adaptation and validation of the newest vital sign in The Netherlands. *Patient Educ. Couns.* **2014**, *97*, 403–409, doi:10.1016/j.pec.2014.08.017.

11. Hoffman, S.; Trout, A.L.; Nelson, T.D.; Huscroft-D 'angelo, J.; Sullivan, J.; Epstein, M.H.; Gibbons, C. A Psychometric Assessment of Health Literacy Measures among Youth in a Residential Treatment Setting. **2013**, *5*, 288–300.
12. Hoffman, S.; Marsiglia, F.F.; Lambert, M.C.; Porta, M. A psychometric assessment of the Newest Vital Sign among youth in Guatemala city. *J. Child Adolesc. Behav.* **2015**, *3*, 190, doi:10.4172/2375-4494.1000190.
13. Howe, C.J.; Van Scoyoc, C.; Alexander, G.K.; Stevenson, J.L. Poor Performance of Children Age 7 to 13 Years on the Newest Vital Sign. *HLRP Heal. Lit. Res. Pract.* **2018**, *2*, e175–e179, doi:10.3928/24748307-20180830-01.
14. Huang, Y.M.; Shiyanbola, O.O.; Smith, P.D.; Chan, H.Y. Quick screen of patients' numeracy and document literacy skills: The factor structure of the newest vital sign. *Patient Prefer. Adherence* **2018**, *12*, 853–859, doi:10.2147/PPA.S165994.
15. Kirk, J.K.; Grzywacz, J.G.; Arcury, T.A.; Ip, E.H.; Nguyen, H.T.; Bell, R.A.; Saldana, S.; Quandt, S.A. Performance of health literacy tests among older adults with diabetes. *J. Gen. Intern. Med.* **2011**, *27*, 534–540, doi:10.1007/s11606-011-1927-y.
16. Kogure, T.; Sumitani, M.; Suka, M.; Ishikawa, H.; Odajima, T.; Igarashi, A.; Kusama, M.; Okamoto, M.; Sugimori, H.; Kawahara, K. Validity and reliability of the Japanese version of the Newest Vital Sign: A preliminary study. *PLoS One* **2014**, *9*, e94582, doi:10.1371/journal.pone.0094582.
17. Kordovski, V.; Woods, S.; Avci, G.; Verduzco, M.; Morgan, E. Is the Newest Vital Sign (NVS) a Useful Measure of Health Literacy in HIV Disease? *Int Assoc Provid AIDS Care* **2017**, *16*, 595–602, doi:10.1177/2325957417729753.
18. Manganello, J.A.; DeVellis, R.F.; Davis, T.C.; Shottler-Thal, C. Development of the Health Literacy Assessment Scale for Adolescents (HAS-A). *J. Commun. Healthc.* **2015**, *8*, 172–184, doi:110.1016/j.bbi.2017.04.008.
19. Mansfield, E.D.; Wahba, R.; Gillis, D.E.; Weiss, B.D.; L'Abbé, M. Canadian adaptation of the Newest Vital Sign©, a health literacy assessment tool. *Public Health Nutr.* **2018**, *21*, 2038–2045, doi:10.1017/S1368980018000253.
20. Martins, A.C.; Andrade, I.M. Cross-cultural adaptation and validation of the Portuguese version of the Newest Vital Sign. *Rev. Enferm. Ref.* **2014**, *IV*, 75–83, doi:10.1016/j.knee.2011.04.006.
21. Miser, W.F.; Jeppesen, K.M.; Wallace, L.S. Clinical utility of a brief screen for health literacy and numeracy among adults with diabetes mellitus. *Fam. Med.* **2013**, *45*, 417–423.
22. Morrison, A.K.; Schapira, M.M.; Hoffmann, R.G.; Brousseau, D.C. Comparison of the Newest Vital Sign and S-TOFHLA. **2014**, *53*, 1264–1270, doi:10.1177/0009922814541674.
23. Norrafizah, J.; Asiah, M.; Suraiya, S.; Zawaha, H.; Normawati, A.; Farid, B.; Faizal, B.; Nasir, A. Assessment of Health Literacy among People in a Rural Area in Malaysia Using Newest Vital Signs Assessment. *Br. J. Educ. Soc. Behav. Sci.* **2016**, *16*, 1–7, doi:10.9734/bjesbs/2016/25737.
24. Osborn, C.Y.; Weiss, B.D.; Davis, T.C.; Skripkauskas, S.; Rodrigue, C.; Bass, P.F.; Wolf, M.S. Measuring adult literacy in health care: Performance of the newest vital sign. *Am. J. Health Behav.* **2007**, *31*, doi:10.5993/ajhb.31.s1.6.
25. Ozdemir, H.; Alper, Z.; Uncu, Y.; Bilgel, N. Health literacy among adults: A study from Turkey. *Health Educ. Res.* **2010**, *25*, 464–477, doi:10.1093/her/cyp068.
26. Paiva, D.; Silva, S.; Severo, M.; Moura-Ferreira, P.; Lunet, N.; Azevedo, A. Limited health literacy in Portugal assessed with the Newest Vital Sign. *Acta Med. Port.* **2017**, *30*, 861–869.
27. Ramirez-Zohfeld, V.; Rademaker, A.W.; Dolan, N.C.; Ferreira, M.R.; Eder, M.M.; Liu, D.; Wolf, M.S.; Cameron, K.A. Comparing the Performance of the S-TOFHLA and NVS among and between English and Spanish Speakers. *J. Health Commun.* **2015**, *20*, 1458–1464, doi:10.1080/10810730.2015.1018629.
28. Rodrigues, R.; De Andrade, S.M.; González, A.D.; Birolim, M.M.; Mesas, A.E. Cross-cultural adaptation and validation of the Newest Vital Sign (NVS) health literacy instrument in general population and highly educated samples of Brazilian adults. *Public Health Nutr.* **2017**, *20*, 1907–1913, doi:10.1017/S1368980017000787.
29. Rowlands, G.; Khazaezadeh, N.; Oteng-Ntim, E.; Seed, P.; Barr, S.; Weiss, B.D. Development and validation of a measure of health literacy in the UK: The Newest Vital Sign. *BMC Public Health* **2013**, *13*, 116, doi:10.1186/1471-2458-13-116.
30. Russell, A.M.; Patel, D.A.; Curtis, L.M.; Kim, K.Y.A.; Wolf, M.S.; Rowland, M.E.; McCarthy, D.M. Test-retest reliability of the Newest Vital Sign health literacy instrument: In-person and remote administration. *Patient Educ. Couns.* **2019**, *102*, 749–752, doi:10.1016/j.pec.2018.11.016.
31. Tseng, H.-M.; Liao, S.-F.; Wen, Y.-P.; Chuang, Y.-J. Adaptation and validation of a measure of health

literacy in Taiwan: The Newest Vital Sign. *Biomed. J.* **2018**, *41*, 273–278, doi:10.1016/j.bj.2018.07.001.
32. Warsh, J.; Chari, R.; Badaczewski, A.; Hossain, J.; Sharif, I. Can the Newest Vital Sign Be Used to Assess Health Literacy in Children and Adolescents? *Clin. Pediatr. (Phila).* **2014**, *53*, 141–144, doi:10.1177/0009922813504025.
33. Weiss, B.D.; Mays, M.Z.; Martz, W.; Castro, K.M.; DeWalt, D.A.; Pignone, M.P.; Mockbee, J.; Hale, F.A. Quick assessment of literacy in primary care: The Newest Vital Sign. *Ann. Fam. Med.* **2005**, *3*, 514–522, doi:10.1370/afm.405.
34. Wolf, M.S.; Curtis, L.M.; Wilson, E.A.H.; Revelle, W.; Waite, K.R.; Smith, S.G.; Weintraub, S.; Borosh, B.; Rapp, D.N.; Park, D.C.; et al. Literacy, cognitive function, and health: Results of the LitCog study. *J. Gen. Intern. Med.* **2012**, *27*, 1300–1307, doi:10.1007/s11606-012-2079-4.
35. Xue, J.; Liu, Y.; Sun, K.; Wu, L.; Liao, K.; Xia, Y.; Hou, P.; Xue, H.; Shi, H. Validation of a newly adapted Chinese version of the Newest Vital Sign instrument. *PLoS One* **2018**, *13*, e0190721, doi:10.1371/journal.pone.0190721.
36. Zotti, P.; Cocchi, S.; Polesel, J.; Mis, C.C.; Bragatto, D.; Cavuto, S.; Conficconi, A.; Costanzo, C.; De Giorgi, M.; Drace, C.A.; et al. Cross-cultural validation of health literacy measurement tools in Italian oncology patients. *BMC Health Serv. Res.* **2017**, *17*, 410, doi:10.1186/s12913-017-2359-0.

Newest Vital Sign | Portuguese adolescent population version (NVS-PTeen)

Esta informação encontra-se no verso de uma embalagem de 0,5L de gelado

**Informação nutricional**		
Cada porção tem	125 mL	
Porções por embalagem	4	
**Quantidades por porção**		
Calorias: 250 cal	Calorias provenientes de gordura: 120	
		**%VDR***
**Teor total de gordura**	13 g	20%
Gorduras saturadas	9 g	40%
**Colesterol**	28 mg	12%
**Sódio**	55 mg	2%
**Teor total de hidratos de carbono**	30 g	12%
Fibra alimentar	2 g	
Açúcares	23 g	
**Proteínas**	4 g	8%

*As percentagens do valor diário recomendado (VDR) são baseadas numa dieta de 2000 calorias diárias. Os seus valores diários podem ser mais baixos ou mais elevados dependendo das suas necessidades calóricas.

Ingredientes: Natas, leite magro, xarope, água, gemas de ovo, açúcar mascavado, gordura do leite, óleo de amendoim, açúcar, manteiga, sal, carragenina, extracto de baunilha.

PERGUNTAS

1. Se comeres a embalagem inteira, quantas calorias estarás a consumir? ....

2. Se te for permitido consumir 60 gramas de hidratos de carbono numa sobremesa, que quantidade de gelado podes comer? ....

3. O teu médico aconselhou-te a reduzir a quantidade de gorduras saturadas na tua alimentação. Habitualmente, consomes 42 gramas de gorduras saturadas por dia, já contando com uma porção de gelado. Se deixares de comer gelado, quantos gramas de gorduras saturadas passarás a consumir por dia? ....

4. Se habitualmente consumires 2500 calorias por dia, que percentagem desse valor é que estarás a consumir se comeres uma porção de gelado? ....

Supõe que és alérgico às seguintes substâncias: penicilina, amendoins, látex e picadas de abelha.

5. É seguro para ti comeres este gelado? ....

6. Se não, por que não? ....

Correct answers

Question 1 : *1000 kcal*
Question 2 : *Up till two portions (inclusively)*
Question 3 : *33 g*
Question 4 : *10%*
Question 5 : *No (not safe)*
Question 6 : *Because it contains peanuts*

Scoring

The overall score of the NVS is obtained with a linear sum of the correct answers (each valuing as 1). *Likelihood of inadequate health literacy* corresponds to: 0 to 1 correct answers; *Limited health literacy* corresponds to 2 to 3 correct answers; and *adequate health literacy* corresponds to 4 to 6 correct answers.
